# The Challenges of Being a Family Caregiver During the COVID-19 Pandemic in Canada

**DOI:** 10.1093/geroni/igab046.2110

**Published:** 2021-12-17

**Authors:** Gwen McGhan, Deirdre McCaughey

## Abstract

The COVID-19 pandemic has impacted all of our lives, but the population most at risk are older adults. Canadians over the age of 60 account for 36% of all COVID-19 cases but 95% of the deaths, and over two-thirds of ICU admissions. Older adults with chronic health conditions are especially at risk. Prior to COVID-19, family caregivers (FCGs) for older adults were managing their caregiving duties at the limits of their emotional, physical and financial capacity. As such, FCGs need special consideration during these times of uncertainty to support them in their role and enable the continuation of care for their older adult family members. This symposium will report on independently conducted studies from across Canada that have examined how the pandemic and associated public health measures have influenced resource utilization by FCGs and the older adults for whom they provide care. McAiney et al’s study examines the deleterious effect of reduced services on community dwelling FCGs and the wellbeing of their family member with dementia. Parmar & Anderson examined the effect of pandemic restrictions on FCGs of frail older adults and found they were experiencing increased distress and decreased wellbeing. Flemons et al report on the experiences of FCGs managing caregiving without critical services and the effect of restrictive visiting policies and the well-being of the caregiving dyad (FCGs and family member with dementia). Finally, McGhan et al will share how FCGs evaluated the efficacy of public health measures and the public health messaging about the pandemic.

